# Supplementary material for: An associative memory Hamiltonian model for DNA and nucleosomes
Source: PLoS Comput Biol. 2023 Mar 27;19(3):e1011013. doi: 10.1371/journal.pcbi.1011013 (PMC10079229; doi:10.1371/journal.pcbi.1011013)
Supplement: S1 Document — Detailed description of the reduced unit system, force field setup, and mechanical property analysis for WEChroM systems.ik (DOCX) [file pcbi.1011013.s001.docx]

# Supporting information

**Appendix A. Simulation with Reduced Units.** In the WEChroM simulations, we use the dimensionless reduced unit system where the unit of length, the unit of energy, and the unit of mass are set to 1.0. When converting between the standard units and the reduced units, we set half of the equilibrium distance between the center of mass of two adjunct nucleotides on the same strand (estimated to be 0.241 nm in standard units) as one unit of length, the mass of one nucleotide (averaged to be 325 a.u. in standard units) as one unit of mass, and $k_{B}T$ at room temperature (300K in standard units) as the unit of energy.

**Appendix B. Connectivity Interactions and Steric Interactions.**

The two basic polymer parts of the potential energy are the connected-chain terms $U_{con}$ and steric terms $U_{vol}$. The connected-chain term *U_con_* is a harmonic spring between the nearest particles on the same strand given by

$$U_{con}=\frac{1}{2}k_{con}\left( r_{i, i+1}-r_{0} \right)^{2}$$

( 9 )

where $r_{i, i+1}$ is the distance between the nearest particles on the same strand, $k_{con}$ is the harmonic force constant, and r_0_ is the equilibrium position distance between two particles and is set to 2.0 in the reduced unit system. It plays the role of forming backbone covalent bonds between the two neighbor nucleotides which should not break under room temperature.

The steric energy *U_vol_* is short-range repulsive energy between any pair of particles to prevent them from overlapping with each other, excepting the pair of the two nearest particles on the same strand, which are already constrained by the harmonic springs of *U_con_*. It is given by

$$U_{vol}=k_{vol}\left( r_{ij}-r^{*} \right)^{2}H(r^{*}-r_{ij})$$

( 10 )

where $k_{vol}$ is the harmonic force constant, $r_{ij}$ is the distance between particle *i* and particle *j* to which the energy is applied, and *r^*^* is approximately twice the effective radius of the particle and is set to be 2.07, *H(x)=1* for *x>0*, *H(x)=0* otherwise.

These two energies form the "backbone" of our DNA system, and are meant to preserve polymer connectivity in single-stranded DNA. It is clear that *U_con_* should be much larger than the thermal energy *k_B_T* when $\Delta r=\left( r-r_{0} \right)$ is comparable to *r_0_*, because in practice the DNA single chain can hardly be broken at room temperature due to the covalent bond connecting the nucleotides. In our model, we set the *k_con_* on such a scale that when $\Delta r\sim0.01r_{0}$, *U_con_* is comparable to *k_B_T*. Accordingly, our relative bond length variance between the nearest particles is $\sim0.01$, consistent with previous reports and simulations(1). Detailed parameters are provided in S1 Table.

**Appendix C. Comparison between WEChroM and Open3SPN2.**

As a more coarse-grained model, our model gives better computing efficiency than that of 3SPN.2(1). We performed simulations of 250-bp naked DNA supercoiling for 24 hours based on the same environment (NVIDIA Tesla V100, CUDA version 10.1, OpenMM version 7.4) using these two models. Our model executed 1.02e9 steps while the 3SPN model executed 5.4e7 steps, a nearly 20 fold increase in efficiency in terms of execution steps.

**Appendix D. Calculations of the linking number, writhing number, and twisting number.**

The linking number describes the number of times that one curve winds around the other. Mathematically, the linking number of two closed curves *C_1_* and *C_2_* is

$$Lk= \frac{1}{4\pi}\int_{C1} \int_{C2} d\Omega(\boldsymbol{r}_{1}, \boldsymbol{r}_{2})$$

( 11 )

where ***r****_1_* and ***r****_2_* are two arbitrary points passing along the curves *C_1_* and *C_2_* and the solid angle *dΩ(****r****_1_,* ***r****_2_)* is defined by

$$d\Omega\left( \mathbf{r}_{\mathbf{1}}, \mathbf{r}_{\mathbf{2}} \right)= \frac{\left( d\boldsymbol{r}_{2}\times d\boldsymbol{r}_{1} \right)\boldsymbol{r}_{12}}{r_{12}^{3}}$$

( 12 )

where *d****r****_1_* and *d****r****_2_* are two infinitesimal vectors originating from ***r****_1_* and ***r****_2_*, the vector ***r***_12_ = ***r***_2_ − ***r***_1_, and *r*_12_ = |***r***_12_|. The writhe of a curve C measures the number of times that one curve winds around itself, and the mathematical formula is

$$Wr= \frac{1}{4\pi}\int_{C} \int_{C} d\Omega(\boldsymbol{r}_{1}, \boldsymbol{r}_{2})$$

( 13 )

where *dΩ(****r****_1_,* ***r****_2_)* is defined by Eq. 12 and *d****r****_1_* and *d****r****_2_* pass along the curve *C*.

The Gauss double integral (Eq. 11) along a DNA polygon of *N* segments can be expressed as the double sum,

$$Lk=\sum_{i=1}^{N} \sum_{j=1}^{N} \frac{\Omega_{ij}}{4\pi}$$

( 14 )

where *i* and *j* are the DNA particles on the two strands respectively. The writhing number in the DNA system is

$$Wr=2\sum_{i=1}^{N} \sum_{j>i}^{N} \frac{\Omega_{ij}}{4\pi}$$

( 14 )

where *i* and *j* pass along the centerline of the DNA, defined by each base pair center of mass. To calculate the solid angle *Ω_ij_* in the DNA system, we use method 1a provided by Konstantin Klenin and Jörg Langowski(2). The twisting number measures the number of rotations the DNA polymer possesses and can be derived by $Tw = Lk - Wr$.

**Appendix E. WEChroM Software Tutorial.**

**E.1 General Usage**

We provide a description for general usage here. It might be helpful to learn the usage of the two examples in the following section S5.2 [Example 1: Naked DNA](https://wechrom.readthedocs.io/en/latest/tutorial.html#example-1-naked-dna).

After installing the wechrom package, you should be able to import the modules by

**import** **wechrom**

We provide two classes, WechromSystem for DNA-only systems and SingleNucleoSystem for single nucleosomes. To initiate the system, you need to provide a pdbx/mmcif file or a pdb file to set up the topology.

your_system = WechromSystem(your_cif_file) *# vanilla DNA system*

or

your_system = SingleNucleoSystem(your_cif_file) *# single nucleosome system*

The default forces designed in WEChroM can be applied by

your_system.addDefaultForces()

You can also add any custom forces supported by openmm

your_system.addForce(your_custom_force, "name_of_your_force")

At this stage, you can make use of your_system.topology, your_system.system to set up your openmm simultions, or you can also initialize the simulation with default langevin integrator and wechrom topology with our method

your_system.initializeSimulation(platform = 'CPU') *# platform can be 'CPU', 'CUDA' or 'OpenCL' depending on your openmm installation*

After simulation initialization, you can make use of your_system.simulation, your_system.integrator to run simulations, or you can use our default dcd reporter and energy reporter to run the simulations

your_system.runSteps(steps = your_steps, reportFreq = your_reportFreq)

**E.2 Example 1: Naked DNA**

This section will instruct you to build a vanilla wechrom system (DNA only) and run simulations. You can find the example notebook at wechrom/examples/naked_75bp/naked_75bp.ipynb. You can run this example interactively using the Jupyter notebook.

After installing the wechrom package, you should be able to import the modules, classes and functions from the package. In this example, we utilize the WechromSystem class.

**from** **wechrom** **import** WechromSystem

We have prepared a pdbx to set up the system. It’s a corase grained 75-bp naked DNA molecule. You can coarse grain an atomistic DNA pdb file into a wechrom cif file with our utility function.

**from** **wechrom** **import** coarse_grain_atompdb_2_wechromcif

*# a cif file will be generated at your working directory*

coarse_grain_atompdb_2_wechromcif(YOUR_PDB_FILE, out_dir = os.getcwd(), out_file_prefix = 'coarse_grain')

Initialize the wechrom system with a pdbx/mmcif file

naked_75bp = WechromSystem("naked_75bp.cif", verbose = **True**)

Apply default forces designed for wechrom

naked_75bp.addDefaultForces()

Output with verbose:

Building connectivity terms...... done

Building excluded volume force...... done

Preparing the associative memory files...... done

Building intra-strand associative memory force...... done

Building inter-strand associative memory force...... done

Initialize the simulation with default integrator and wechrom topology

naked_75bp.initializeSimulation()

Output with verbose:

Langevin integrator **and** simulation initialized

Run simulation with trajectory and energy reported.

naked_75bp.runSteps(steps = 1000, reportFreq = 100)

Output with verbose:

Simulation will take 1000 steps and get reported every 100 steps

----------------Simulation Starts----------------

100%|██████████| 10/10 [00:02<00:00, 4.95it/s]

Simulation done.

Please check your trajectory file movie.dcd, energy file energy.txt at your output directory your_path\examples\naked_75bp

**E.3 Example 2: Single nucleosome**

This section will instruct you to build a single nucleosome wechrom system and run simulations. You can find the example notebook at wechrom/examples/nucleosome_223bp/nucleosome_223bp.ipynb. You can run this example interactively using the Jupyter notebook.

After installing the wechrom package, you should be able to import the modules, classes and functions from the package. In this example, we utilize the SingleNucleoSystem class.

**import** **wechrom**

We have prepared a pdbx to set up the system.Initialize the nucleosome system with a pdbx file we prepared with 147 bp wrapped DNA, 38 bp linker DNA on each end and a histone core particle. This cif file also includes two virtual sites at the two ends for external force illustration.

nuc_223bp = wechrom.SingleNucleoSystem("singleN_L38_endvs.cif", verbose = **True**)

Apply default forces designed for wechrom

nuc_223bp.addDefaultForces()

Output with verbose:

Building connectivity terms...... done

Building excluded volume force...... done

Preparing the associative memory files...... done

Building DNA intra-strand associative memory force...... done

Building DNA inter-strand associative memory force...... done

Preparing the nucleosome associative memory files...... done

Building nucleosome center associative memory force...... done

Building nucleosome neighbor associative memory force...... done

Here we provide an example of applying external force to the system. The desired external force is to pull the two ends apart in x direction. You may use any force supported by openmm

**from** **openmm** **import** CustomExternalForce

**def** stretch_term(we, k_stretching=2.0, forceDirect="x", appliedSite=0):

stretching = CustomExternalForce(f"(*{*k_stretching*}*)*(*{*forceDirect*}*)")

stretching.addParticle(we.virtualSites[appliedSite])

**return** stretching

nuc_223bp.addForce(stretch_term(nuc_223bp, forceDirect="x", appliedSite=0), "pull_h")

nuc_223bp.addForce(stretch_term(nuc_223bp, forceDirect="-x", appliedSite=-1), "pull_t")

Initialize the simulation with default integrator and wechrom topology

nuc_223bp.initializeSimulation(platform='CUDA')

Output with verbose:

Langevin integrator **and** simulation initialized

Here we provide an example of adding a pdb reporter to the simulation

from openmm.app import PDBReporter # openmm version >= 7.6 # from simtk.openmm.app import PDBReporter # openmm version < 7.6 steps = 1e3 reportFreq = 1e1 nuc_223bp.simulation.reporters.append(PDBReporter(“./movie.pdb”, reportFreq

Run simulation with trajectory and energy reported.

nuc_223bp.runSteps(steps = steps, reportFreq = reportFreq)

Output with verbose:

Simulation will take 1000 steps and get reported every 100 steps

----------------Simulation Starts----------------

100%|██████████| 10/10 [00:02<00:00, 38.37it/s]

Simulation done.

Please check your trajectory file movie.dcd, energy file energy.txt at your output directory your_path\examples\nucleosome_223bp


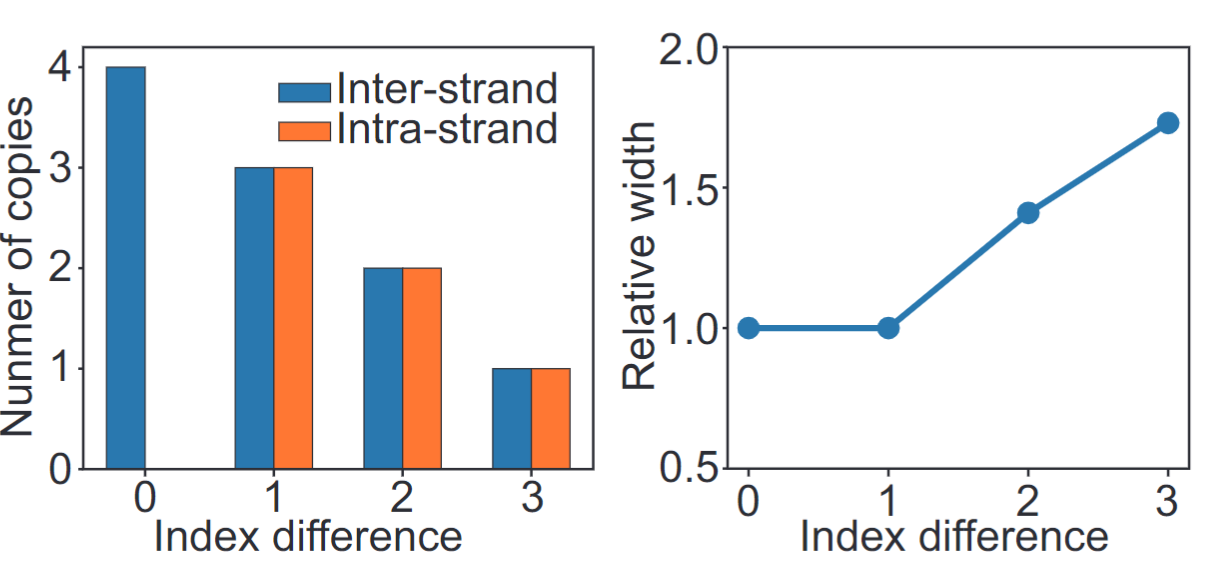


**Fig A. Counting and the relative variance** $\boldsymbol{\sigma}$ **of associative memory terms.** (a) Due to the nature of counting, the number of associative memory bonds applied to a pair of particles depends on the index difference. (b) We expand the relative variance $\sigma$ of the bonds depending on the index difference in the trend of $\sqrt{index difference}$


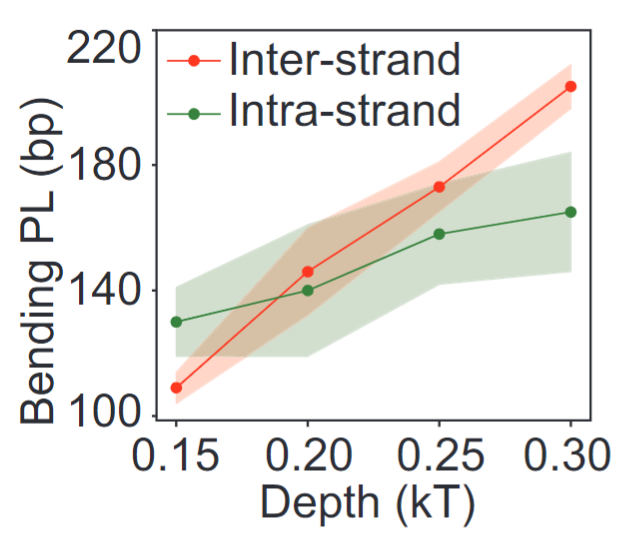


**Fig B. Model flexibility.** The figure shows how bending persistence lengths depend on the two types of energy scaling factor $\lambda$. The solid lines are persistence length for inter-strand (red) $\lambda$ and intra-strand factor $\lambda$ (green) and the shaded area are standard error. The variance $\sigma$ is fixed at 0.15. In each line, only one $\lambda$ is varied and the other is fixed.


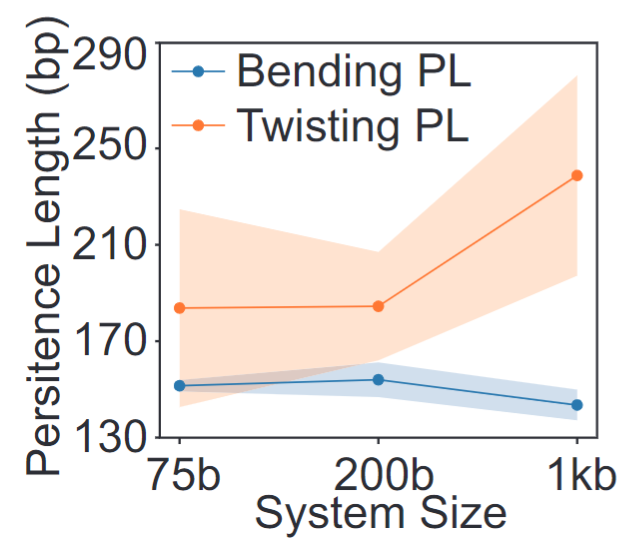


**Fig C. Systems of different sizes are consistent in persistence length.** The figure shows how twisting and bending persistence lengths depend on the size of the WEChroM system. The solid lines are persistence lengths for bending (blue) and twisting (orange) on different system sizes and the shaded area is the standard error.

| Parameter (unit) | Value |
| --- | --- |
| *k_con_* (*k_B_T*) | 3000 |
| *r_0_* | 2.0 |
| *k_vol_* (*k_B_T*) | 5856 |
| *r** | 2.07 |

**Table A. Parameters in** $\boldsymbol{U}_{\boldsymbol{con}}$ **and** $\boldsymbol{U}_{\boldsymbol{vol}}\boldsymbol{.}$We used the reduced units, where the equilibrium distance between two adjunct particles on the same strand is 2.0 (around 0.482 nm in standard units). The energy is expressed in the unit of $k_{B}T$, where the temperature ($T$) of the simulation is fixed and assumed to be room temperature, and we tune the energy terms relatively. The parameters used in $U_{con}$ and $U_{vol}$ are summarized in the table.

| λ (*k_B_T*) | σ (r.u) | *L_bp_* (bp) | *L_tp_* (bp) |
| --- | --- | --- | --- |
| 0.15 | 0.05 | *144* | *188* |
| 0.15 | 0.1 | 153 | 172 |
| 0.2 | 0.15 | 140 | 191 |
| 0.25 | 0.17 | 154 | 196 |
| 0.3 | 0.2 | 151 | 219 |

**Table B. Multiple Sets of Parameters in** $\boldsymbol{U}_{\boldsymbol{DH}}$**.** These sets of parameters all give a bending persistence length *L_bp_* ~ 150 bp.

# References

1. Hinckley DM, Freeman GS, Whitmer JK, de Pablo JJ. An experimentally-informed coarse-grained 3-site-per-nucleotide model of DNA: Structure, thermodynamics, and dynamics of hybridization. J Chem Phys. 2013 Oct 14;139(14):144903.

2. Klenin K, Langowski J. Computation of writhe in modeling of supercoiled DNA. Biopolymers. 2000;54(5):307–17.
